# P21.1 at PETRA III – a high-energy X-ray diffraction beamline for physics and chemistry

**DOI:** 10.1107/S1600577525002826

**Published:** 2025-04-22

**Authors:** Martin v. Zimmermann, Oleh Ivashko, Fernando Igoa Saldaña, Jiatu Liu, Philipp Glaevecke, Olof Gutowski, Rüdiger Nowak, Katharina Köhler, Björn Winkler, Andreas Schöps, Horst Schulte-Schrepping, Ann-Christin Dippel

**Affiliations:** a Deutsches Elektronen-Synchrotron DESY Notkestr. 85 22607Hamburg Germany; b Goethe Universität Frankfurt Institut für Geowissenschaften Kristallographie/Mineralogie Altenhöferallee 1 60438Frankfurt am Main Germany; RIKEN SPring-8 Center, Japan

**Keywords:** high-energy X-rays, X-ray diffraction, total scattering, single-crystal diffuse scattering

## Abstract

Beamline P21.1 at PETRA III uses high-energy photons for the investigation of materials structure by diffraction methods. The instrumentation is particularly suited for probing ordering phenomena on a local scale in chemistry and physics. A detailed description of the instrumentation and detectors for such experiments is given. The beamline supports a number of sample environments for investigations under *in situ* and *operando* conditions, *e.g.* cryostats, furnaces, chemical reactors. Recent examples of measurements on amorphous, single-crystalline and thin film samples are described.

## Abstract 2

A detailed description of the technical specifications of beamline P21.1 at the PETRA III storage ring is given. It serves as a reference for the beamline user community and, by giving a number of scientific examples, we hope to inspire future experiments.

## 1. Introduction

PETRA III at DESY (Hamburg, Germany) is one of the few high-energy (6 GeV) storage rings worldwide and operates several beamlines providing high-energy X-rays >50 keV of high brilliance. Owing to the high demand in experimental facilities running in this photon energy range, new high-energy beamlines have been built in the PETRA III extension in the past 5–10 years (Balewski *et al.*, 2011 ▸; Drube *et al.*, 2016 ▸). One of these is beamline P21 whose development was largely driven by the Swedish research community. Sweden operates its own synchrotron radiation source MAX IV in Lund which is a 3 GeV machine optimized for the hard X-ray regime. With their country’s share of PETRA III, Swedish scientists have access to an expanded photon energy range, thus complementing their experimental capabilities. P21 is called the Swedish Materials Science (SMS) beamline and is divided into two branches: the side station P21.1 (co-funded by DESY and Sweden) and the inline station P21.2 (fully funded by Sweden). Whereas P21.2 focuses on diffraction and imaging mainly for engineering material science, P21.1 specializes in scattering and diffraction experiments for fundamental and applied research in physics, chemistry, material science and related disciplines.

Under this scope, beamline P21.1 and the second experimental hutch (EH2) of beamline P07 (Bertram *et al.*, 2016 ▸) are operated in tandem by one team and offer a complementary range of high-energy X-ray diffraction and scattering techniques.

At P21.1 high-energy X-rays are used to measure diffraction and scattering patterns while exploiting at least one of the specific advantages of high photon energies, *i.e.* comparatively small scattering angles for a given momentum transfer *Q*, enabling access to high *Q*, high penetration through thick samples, high-*Z* materials and X-ray windows, and a high scattering cross section with an optimized ratio of Thomson scattering versus Compton scattering and absorption. In particular, *in situ* and *operando* experiments benefit from the possibility to employ bulky sample environments with small exit windows. P21.1 focuses on the application of the total scattering technique to gain information about local order and pair correlations. This is performed on single crystals, as well as on powders, liquids and nano-structured materials. Recently the capabilities have been expanded to amorphous and nanocrystalline thin films that are measured by the highly surface-sensitive grazing-incidence technique (Dippel *et al.*, 2019 ▸). For all types of samples, real-space information is obtained via Fourier transformation of the total scattering patterns. For single crystals high-intensity Bragg spots are separated from diffuse scattering signal which is orders of magnitude weaker and located at and/or in-between the Bragg reflections. This difference is used to obtain the 3D pair distribution function (PDF) of the diffuse scattering (3d-ΔPDF), the autocorrelation function of local order in the sample (Weber & Simonov, 2012 ▸). Analysis of the 1D PDF obtained from randomly oriented samples is well established and makes use of standard software packages (Egami & Billinge, 2003 ▸).

Beamline P21.1 uses a single-bounce bent Laue silicon crystal as monochromator with a fixed scattering angle and is specifically made to provide high photon flux at high X-ray energies. Three different monochromator reflections can be chosen, in order to obtain photon energies of 53, 86 and 101 keV. Most recently, compound refractive lenses have been added to the beamline optics, for focusing on the micrometre scale for diffraction from micrometre-sized samples and for experiments under the grazing-incidence condition.

The experimental hutch at P21.1 offers a versatile setup for a large variety of diffraction and scattering applications from simple *ex situ* measurements to complex and challenging *in situ* and *operando* experiments. The hutch houses a heavy-load sample stage providing sufficient space to install bulky sample environments such as cryostats, reactors or vacuum chambers. A suite of standard equipment available at the beamline for high and low temperature, low scattering background or chemical reactions is complemented by dedicated sample environments designed and brought by the users, *e.g.* levitators, mechanical testing setups or (electro)chemical synthesis cells. The available choice of detectors ranges from fast and large area detectors to high-resolution scanning detectors mounted on a stage that is movable over more than 4 m in sample-to-detector distance. The experiments performed cover numerous research fields in physics, chemistry, materials science and other disciplines. Many of the P21.1 users work in the area of strongly correlated electron materials and study superconductors, magnetic or multiferroic materials under non-ambient conditions, often at low temperature and under pressure and magnetic field, to explore complex phase diagrams. Another significant fraction of the P21.1 beam time is dedicated to the investigation of nanomaterials during growth or operation *e.g.* in catalytic or thermoelectric processes, or temperature-induced transitions like crystallization. Bulk metallic glasses as a particular class of engineering materials, as well as liquids, primarily nanoparticle suspensions and solution species, represent amorphous materials regularly studied at the beamline. Research on energy materials extends from the investigation of individual components such as powder or (thin) layers to functional devices like batteries. These and all other experiments cover aspects of fundamental as well as applied science.

## 2. Technical configuration of the beamline

### 2.1. Source

**Figure 1:**
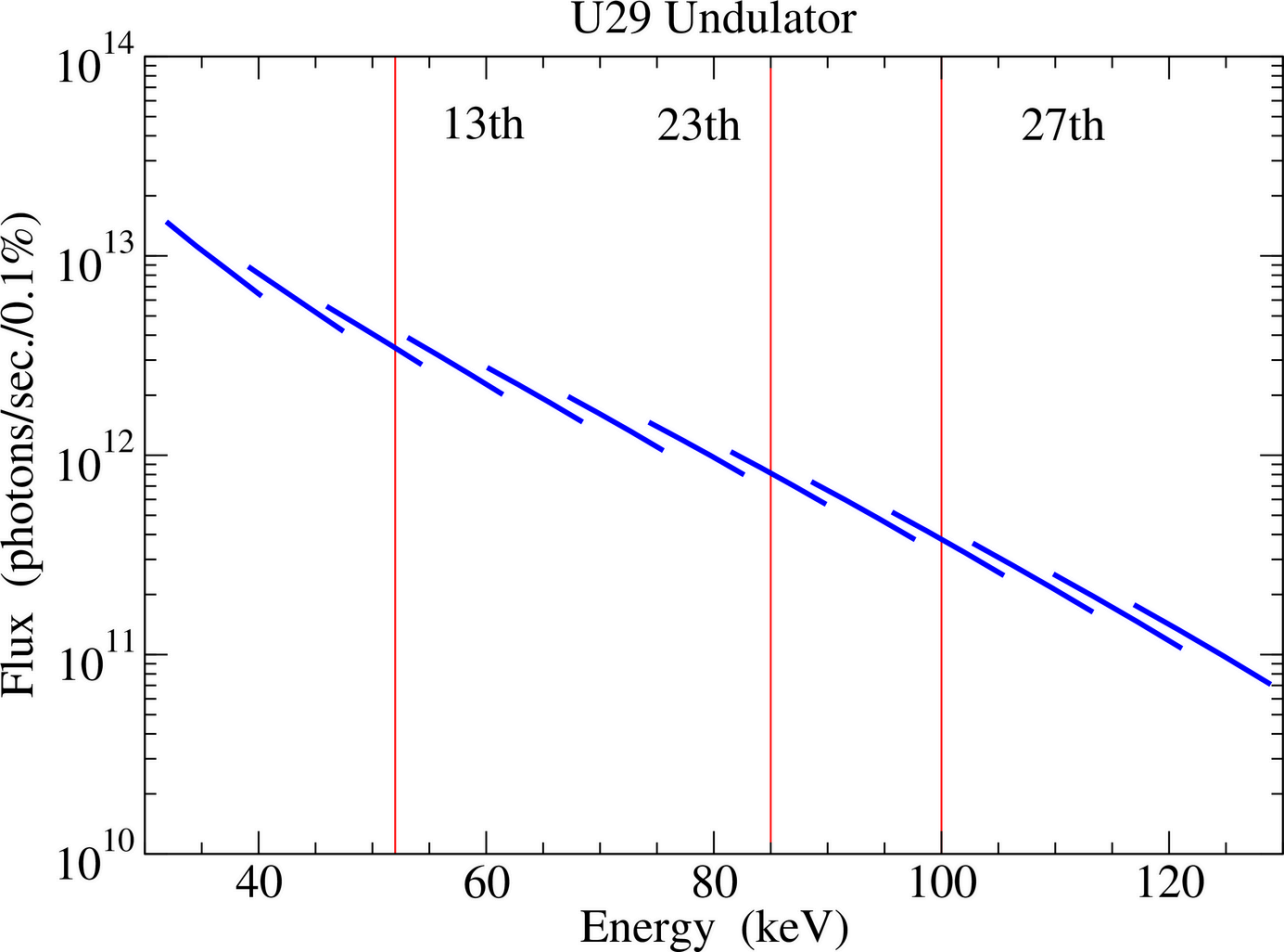
Flux of the U29 undulator through a 1 mm × 1 mm slit at 80 m distance from the source and a bandwidth of 0.1% at beamline P21.1. The available energies are shown by red lines with the corresponding undulator harmonics.

Each of the two branches of the SMS beamline is served by a separate undulator, both located in the long straight section in PETRA III experimental hall east. They are canted by an angle of 1 mrad in order to generate two independent beams separated by 8 cm at 80 m distance from the undulators, which is the location of the entrance into the first optics hutch. At P21.1 a U29 undulator is used with the parameters listed in Table 1 ▸ and the undulator spectrum is shown in Fig. 1 ▸ (Schöps *et al.*, 2016 ▸). Water-cooled power slits are used to shape the beam around the central cone of the beam generated by the undulator. The heat load of the beam onto the optical elements is reduced by utilization of a filter of 50 µm Cu on 300 µm chemical vapour deposition (CVD) diamond which absorbs the low-energy part of the spectrum (Schulte-Schrepping *et al.*, 2016 ▸).

### 2.2. Optics

**Figure 2:**
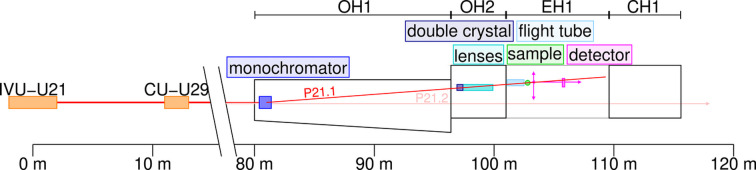
Top view of the P21 front-end and the P21.1 part of the Swedish beamline. Two canted undulators generate two independent beams for the two beamlines. P21.1 uses a single-bounce Laue monochromator in order to accomplish an offset to the in-line experiment of beamline P21.2.

A horizontally scattering bent Laue single-crystal monochromator is used in order to select a specific energy from the undulator spectrum and to accomplish a large offset from the beam tube of beamline P21.2 (see Fig. 2 ▸). The beam of the P21.2 beamline passes through the vacuum vessel of the P21.1 monochromator but does not hit the monochromator crystal. The scattering angle of the monochromator is fixed at 2θ_m_ = 4.3°, which generates a horizontal offset of 1.6 m at the sample position, 21 m downstream of the monochromator crystal. Three different reflections of the monochromator crystal can be selected by rotation around the vertical axis: 111, 220 and 311, leading to photon energies of nominally *E*_photon_ = 53, 86 and 101 keV, respectively. At the 311 reflection the asymmetry angle of the crystal is 39.5°. The water-cooled crystal of 2.5 mm thickness is bent to radii between 10 m and infinity (flat crystal) in order to achieve either high photon flux or high energy resolution. Bending towards the sample also has a focusing effect and, hence, maximizes the flux on the sample (Suortti & Thomlinson, 1988 ▸). In the standard high-flux setting, the energy bandwidth is about 0.2%.

When using 101 keV with the 311 reflection, the contamination of the beam with higher-order harmonics is not detectable. At the 111 and 220 reflections at 53 keV and 86 keV, respectively, strong harmonic contamination of a level of 10^−1^ is detected, making them unsuitable for diffraction experiments. Thus, a secondary vertically scattering double-crystal monochromator is used in optics hutch 2 (OH2) for these energies. It consists of two 5 mm-thick Si(311) thermal gradient crystals with a thermal gradient between 60 and 80°C depending on the chosen energy (Rütt *et al.*, 2010 ▸). The two crystals diffract the beam in opposite directions such that the direction of the beam runs parallel to the direct beam with a vertical offset. This device reduces the harmonic contamination to a level of 3 × 10^−4^ for λ/3 at 53 keV, while λ/2 at 53 keV and 86 keV becomes undetectable. λ/3 at 86 keV is naturally small enough to be undetectable.

For a fast and precise determination of the photon energy a so-called *energiebestimmer* has been implemented in the OH2. It is based on the exact determination of Bragg angles as described by Bond (1960 ▸). The device consists of a 0.6 mm-thick Si(100) wafer, two YAG fluorescence screens located on the left- and right-hand side of the direct beam at a distance of about 20 cm behind the crystal and a camera that observes the X-ray-excited optical fluorescence on the two screens. The crystal is rotated such that the angle between the Si(220) and Si(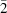

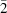
0) reflections scattered on the YAG screen can be precisely measured which is then used to calculate the energy. Beam diagnosis with this device takes about 5 min, runs in an automated fashion, does not require any changes to the sample setup in the experimental hutch and the energy is evaluated from the peak positions of the two reflections by a simple script.

By using 97 Al lenses with 50 µm parabolic radius at a distance of 5.1 m from the sample at a photon energy of *E* = 101 keV a focal size of 2 µm × 150 µm (V × H) is obtained with a flat monochromator crystal. By further relaxing the bending radius, a minimum horizontal focal size of about 80 µm can be achieved at a cost of reducing the intensity by an additional factor of three. The 2 µm × 150 µm setting has been used for grazing-incidence measurements on amorphous thin films as described in Section 3.4. The observed horizontal focus size is significantly larger than typically observed at beamlines with double-crystal monochromators that preserve the source divergence. The larger horizontal focal size at P21.1 stems from the relatively large beam divergence of the single-bounce monochromator, even at large bending radii. A list of photon-beam parameters can be found in Table 2 ▸.

### 2.3. Experimental hutch

**Figure 3:**
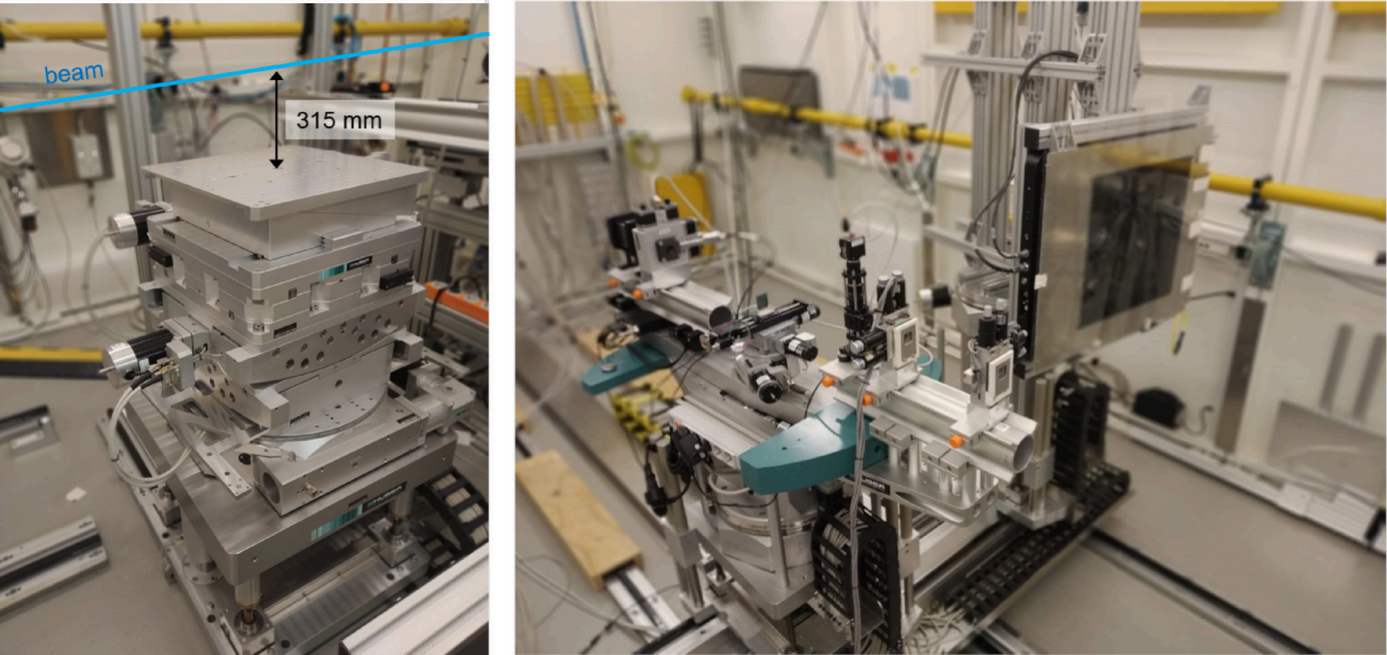
Photo of the sample stage (left) comprising vertical omega axis, double-tilt cradle and *xyz*-translation. Detector stage (right) with independent platforms for large area detector and two-theta arm including optional analyzer crystal.

The heavy-load sample stage shown in Fig. 3 ▸ consists of an *xyz*-translation mounted on top of a double-tilt rotation stage with horizontal axis and a high-precision rotation table with a vertical axis. These are mounted on top of additional coarse vertical and horizontal translations. The top surface of this stage has a size of 400 mm × 400 mm and the center of rotation of the double-tilt stage is 315 mm above the top surface. The sample stage is capable of carrying loads of up to 600 kg. The double-tilt table allows single-crystal experiments with sample tilts up to 7°, depending on the weight of the sample environment. If a larger-angle variation of the sample is needed, a Eulerian cradle can be mounted on top of the sample stage. The latter device is equipped with a motorized *xyz*-translation for a closed-cycle cryostat in order to move the sample in the center of rotation. The ϕ rotation of the Euler circle has a range of 360°.

The detector stage implements the functionality of rapid change between large area detectors for scattering patterns with large *Q*-range coverage and small area and point detectors, for detailed mapping of small sections of momentum space and single reflections, optionally combined with an analyzer crystal to enhance resolution (see Fig. 3 ▸). Both detector types are simultaneously mounted on two platforms on a translation stage whose distance to the sample can be varied from approximately 20 cm to about 4.5 m. One platform is used to position the large area detector. The available flat-panel and hybrid-pixel detectors are described in detail below. The other platform emulates the movement of a two-theta arm by the combination of rotations and translations. The angular travel range in the horizontal is up to about 30° and in the vertical up to 7°, depending on the chosen distance from the sample. The two-theta arm includes a rotation stage for an analyzer crystal mounted on the two-theta rotation, thus naturally maintaining the reflection condition during a two-theta movement. Crystals with different degrees of perfection and reflections can be chosen. When the required angle resolution exceeds the resolution obtained on the area detector, a perfect Si(311) analyzer crystal is used in combination with a point detector, and for suppression of fluorescence and Compton scattering from the measurement signal a SiGe(111) gradient crystal is chosen (Keitel *et al.*, 1999 ▸). Detailed information on the point detectors that can be mounted on the two-theta arm is given below.

An evacuated 1.5 m-long flight tube in front of the sample with an included slit system minimizes air scattering. It can be either directly connected to a sample chamber or extended to a position directly in front of the sample in order to reduce the path length of the direct beam in air and thus the scattering background to a minimum. A beamstop behind the sample serves the same purpose. The intensity of the incoming beam is continuously monitored by measurement of the current of a 300 µm-thick Si diode positioned in the beam in front of the sample.

#### 2.3.1. Detectors

The large area flat-panel detectors available at the beamline are a PerkinElmer XRD1621 and a Varex XRD 4343CT. Their sensor consists of amorphous silicon covered by a CsI scintillator. They are particularly suited for total scattering applications, since these detectors have no gaps between the pixels and scattering data from ‘weakly’ scattering samples are readily integrated. In addition, these detectors have the largest active area of all detectors available at the beamline and hence give access to large momentum transfers.

For strongly scattering samples, however, the flat-panel detectors show significant afterglow, leading to artifacts in the diffraction signal (Siewerdsen & Jaffray, 1999 ▸). A Dectris Pilatus3 X CdTe 2M detector with single-photon counting capability can be requested for specific experiments. It is available in a shared mode amongst high-energy X-ray beamlines at PETRA III. Even though smaller in area, it does not exhibit afterglow and is well suited for fast measurements up to 250 Hz and a dynamic range of 5 × 10^6^ counts per second and per pixel. The two SMS beamlines also share a Dectris Eiger2 X CdTe 4M detector for applications that require particularly fast data acquisition up to 2 kHz or high angular resolution.

As described above, the large area detector can be positioned anywhere behind the sample by using the detector stage. Several modes turn out to be particularly useful: (1) direct beam in the center of the detector, (2) direct beam at the edge of the detector or (3) direct beam in the corner of the detector. For powder diffraction the first setting has the advantage of detecting complete Debye–Scherrer rings, resulting in good statistics and the cancelation of polarization effects in the intensity distribution along the circumference of the ring. With the last method the largest momentum transfer is achieved at a given distance, though at the expense of counting statistics. At P21.1 detector distances routinely range from 300 to 3000 mm and can be expanded by mounting special setups. The dimensions of the available area detectors are listed in Table 3 ▸. A resulting maximum momentum transfer of *Q*_max_ > 20 Å^−1^ as required for high-quality PDF data is readily achieved in case (1) and can be pushed to*Q*_max_ > 30 Å^−1^ in case (3).

For a typical total scattering measurement on capillaries with 1 mm diameter and the PerkinElmer detector positioned in geometry (1) at a sample-to-detector distance of approximately 400 mm, the PDF calibration parameters *Q*_damp_ and *Q*_broad_ (Farrow *et al.*, 2007 ▸) refine to ∼0.04 Å^−1^ and ∼0.01 Å^−1^, respectively, for nickel powder.

Under such conditions the minimum momentum transfer (defined by the beamstop radius and distance to the sample) is about 0.5 Å^−1^. For the largest detector distances, this can be reduced to 0.1 Å^−1^, which is suitable for measuring large coherent distances, such as the ones present in nanoparticle superlattices or typical metal–organic frameworks.

The measurement of single-crystal total scattering is most efficiently performed with the Pilatus detector due to its large dynamic range and negligible afterglow. Data are acquired at three positions shifted in the diagonal direction by about the size of the detector gaps, in order to avoid gaps in the total scattering pattern, which would lead to artifacts in the Fourier transformation. The combination of data sets taken with different incident flux expands the dynamic range even further and avoids artifacts from detector saturation.

The point detectors that can be mounted on the two-theta arm include cyberstar with NaI(Tl) and LaBr_3_(Ce) scintillation sensors as well as an Amptek CdTe diode for experiments using a magnetic field. In combination with a perfect Si crystal analyzer a longitudinal resolution of up to Δ*Q*_∥_/*Q* = 3 × 10^−5^ can be obtained at 101 keV. The transverse resolution is limited by the sample mosaicity and approaches the Darwin width of Δ*Q*_⊥_/*Q* = 1 × 10^−6^. For studies that require high sensitivity to small signals instead of high resolution, analyzer crystals with large reflectivity and tunable rocking-curve width can be used. This includes a SiGe(111) gradient crystal (Keitel *et al.*, 1999 ▸) and a bent crystal analyzer with tunable bending radius which are particularly suited to suppress background from sample fluorescence and Compton scattering. A typical rocking width of the analyzer crystal in this case is 0.1 mrad.

Furthermore, a Pilatus CdTe 100K detector can be used in combination with the two-theta detector arm, which is highly useful to map diffuse scattering within a single Brillouin zone, where the detector sensor is not exposed to the strong Bragg scattering. For the detection of fluorescence Amptek solid-state detectors with Si or CdTe sensors can be used.

### 2.4. Sample environments

A number of sample environments for experiments related to physics and chemistry are available at the beamline. As beamlines P07-DESY and P21.1 are operated by one joint team and the experimental setups at both stations are very similar, the sample environments are mostly compatible with experiments at both beamlines and thus shared for an optimum usage. Table 4 ▸ lists the sample environments available at beamline P21.1.

#### 2.4.1. Scattering chamber

**Figure 4:**
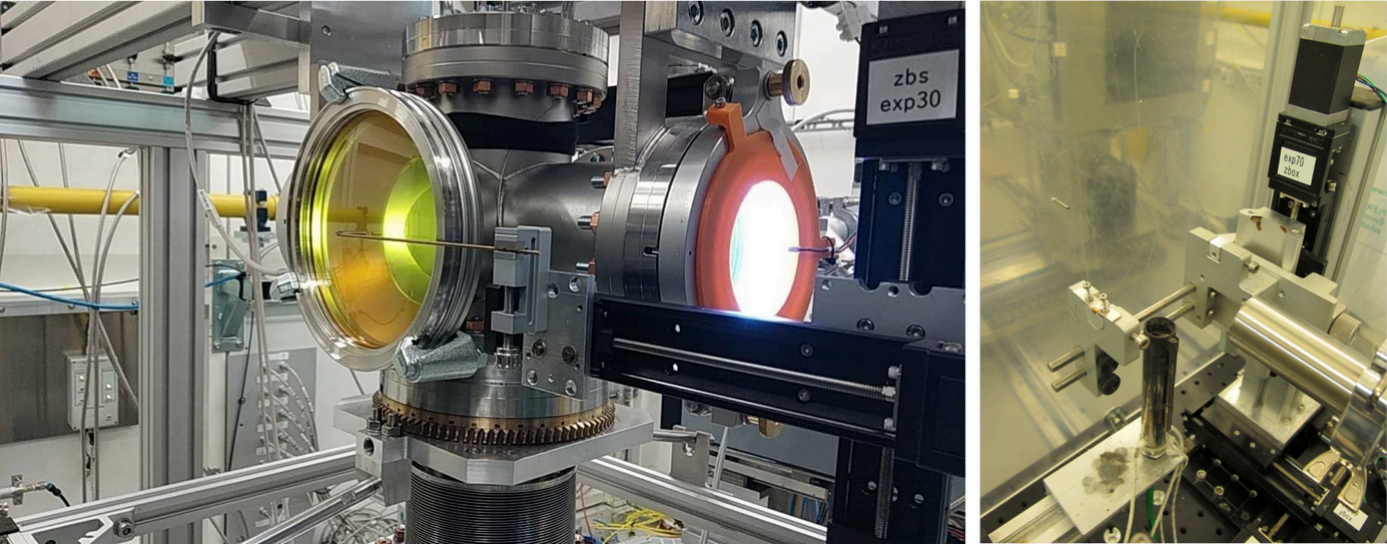
Photo of the scattering chamber (left) and the hot air blower for the chemical reactor inside the splash box (right).

A so-called scattering chamber is available for total scattering experiments on weakly scattering samples and those that need to be kept in vacuum or inert gas atmosphere, as shown in Fig. 4 ▸. It is suited for both randomly oriented as well as single-crystalline samples. Inside the chamber a motorized sample changer for up to 25 capillaries mounted in a frame is used for automated measurement of powders and liquids or other bulk samples. Resistive heating plates made of silicon nitride are available to heat samples in transmission geometry (horizontal or vertical alignment) or in grazing-incidence condition to temperatures up to 1000°C. Single-crystal data sets are taken by rotating the crystal samples via a rotational feedthrough of the sample rotation table into the scattering chamber. This enables a rotation range of 360° around a vertical axis and about 2° around the two horizontal axes. For background suppression the chamber is either evacuated or flooded with helium gas. The background on a hybrid-pixel detector is then less than 1 count per second and pixel in the medium to high *Q* range and and 5 counts per second and per pixel in the low *Q* range. The vacuum system of the scattering chamber is connected directly to the beamline flight path, as described above. A motorized pinhole and beamstop are located inside the chamber in order to avoid background scattering from the entrance and exit windows. The exit window of the chamber has a conical opening angle of 30° and is covered by a glass window. The maximum momentum transfer is *Q*_max_ = 28 Å^−1^ at an energy of 101 keV.

#### 2.4.2. Splash box

For experiments that involve liquids and/or gases under non-ambient conditions that bear the risk of sample spillage, a so-called splash box is available. It consists of a bread-board (dimensions 60 cm × 60 cm) surrounded by Plexiglas walls (80 cm height). The evacuated flight tube connects into the box and flexibly reaches close to the sample position. A beamstop is fixed inside the wall on the exit side and is placed in the beam by moving the entire box by the diffractometer. Translation stages for positioning the sample in all three dimensions parallel and perpendicular to the beam are installed (travel range of 50 mm in each direction). The setup can easily be combined with a hot air blower, *e.g.* for heating capillaries in horizontal orientation such as in the *in situ* cell for solvothermal synthesis described by Roelsgaard *et al.* (2023 ▸).

#### 2.4.3. Displex cryostat

A closed-cycle cryostat for a temperature range between 10 K and 320 K is used either in combination with the Eulerian cradle for detailed scanning in reciprocal space or in combination with the so-called cryo-chamber which allow single-crystal total scattering experiments with continuous rotation of the sample around 360° without contaminating signals from window material, as described below.

**Figure 5:**
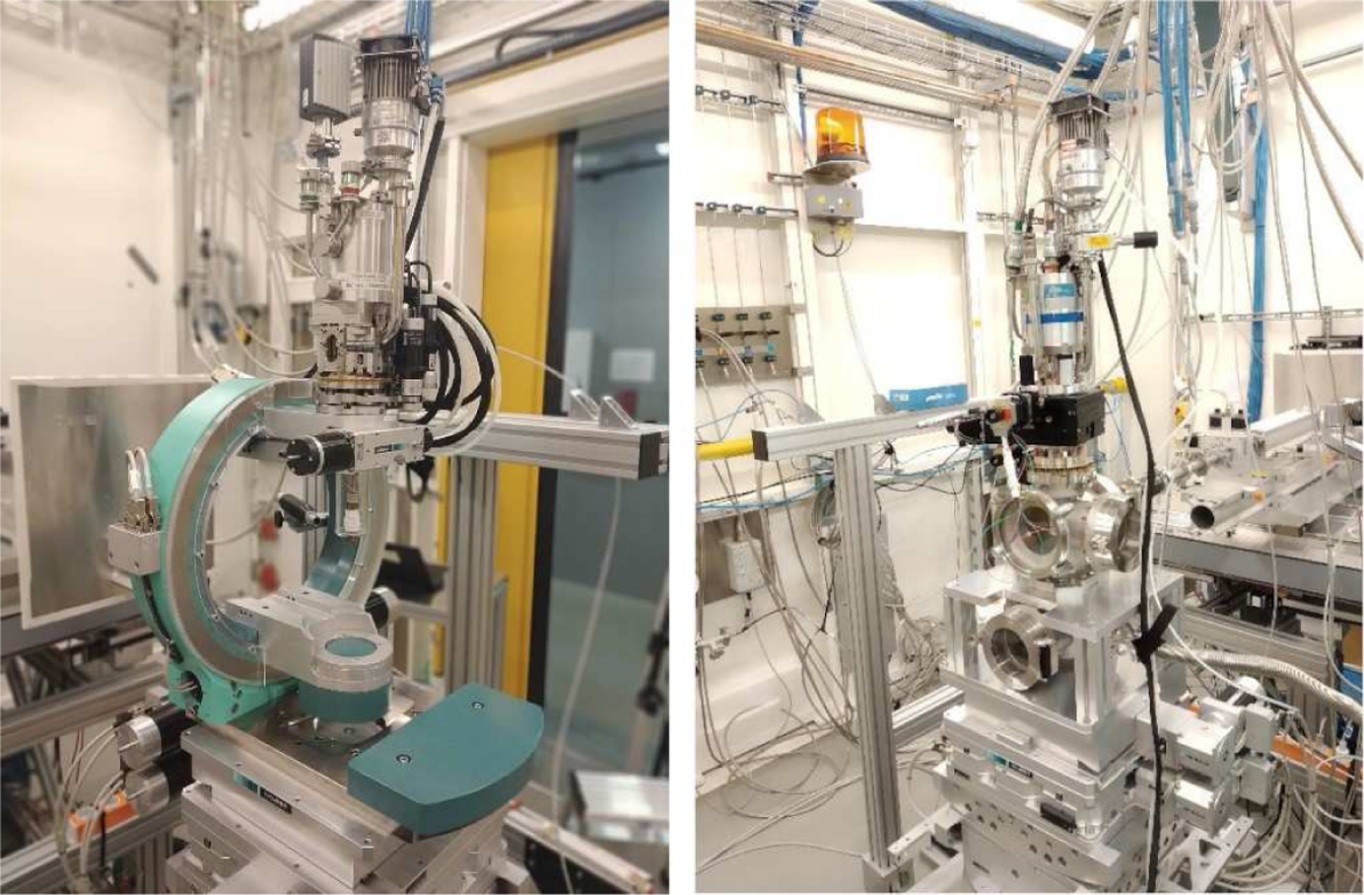
Photo of the displex cryostat mounted in the Eulerian cradle (left) and in the cryochamber (right).

When used with the Eulerian cradle (see Fig. 5 ▸, left) various types of vacuum and radiation shrouds are mounted on the cryostat and *Q* space is scanned in a point detector fashion. Sample centering is performed by a motorized *xyz*-translation. Powder lines from window material (Al) are shielded by employing collimating slits on the scattering side in front of the detector. A combination of Kapton and aluminized Mylar windows is also available.

If the closed-cycle cryostat is used in combination with the cryo-chamber (right-hand side of Fig. 5 ▸), the sample rotation is performed via a rotational feedthrough of the cold finger into the chamber, which is mounted in a fixed position on the sample table. An *xy*-translation allows centering of the sample in the center of rotation. The exit window has a conical opening angle of 35° (*Q*_max_ ≃ 30 Å^−1^ at 100 keV). Single-crystal total scattering patterns can then be measured within a couple of minutes in combination with a large area detector. A radiation shield of aluminized Mylar foil captures the thermal load of the chamber, and adds only little background. Similar to the scattering chamber, the cryo-chamber can be connected to the flight path of the incoming beam. A beamstop positioned inside the chamber in front of the exit window catches the transmitted beam and thus prevents background scattering from the exit window. The cryo-chamber is also suited for powder diffraction at low temperature where a sample holder for three capillaries is available. The limited thermal conductivity of powder samples reduces the temperature range for this type of samples to about 30–320 K.

#### 2.4.4. Helium cryostat

**Figure 6:**
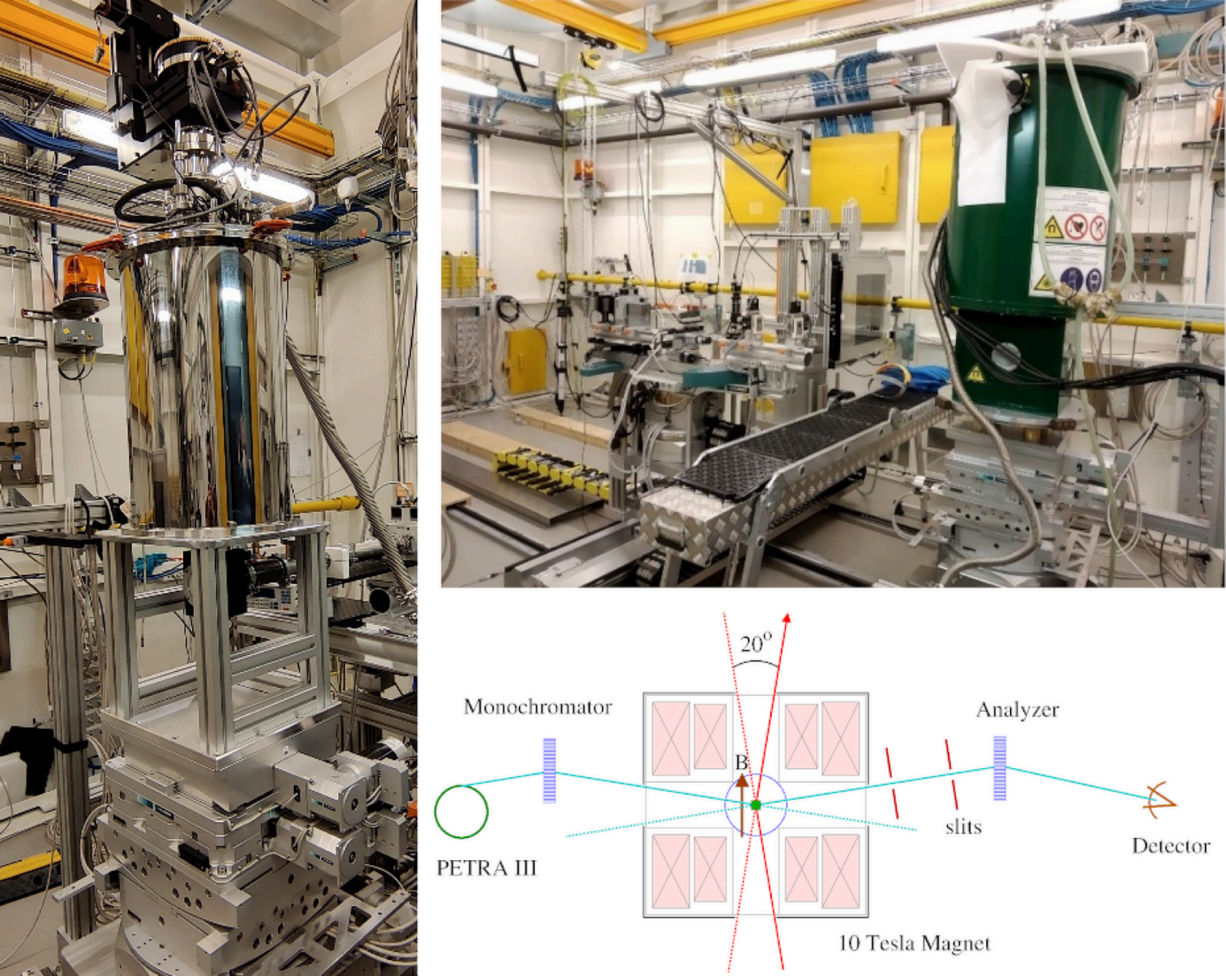
Helium cryostat (left) and 10 T cryomagnet (right) mounted in the experimental hutch of beamline P21.1. The schematic drawing shows the X-ray window arrangement of the cryomagnet.

The accessible temperature range is extended down to*T*_min_ = 1.8 K by utilization of a helium bath cryostat, as shown in Fig. 6 ▸. The maximum temperature of this device is 300 K. It is specifically designed for the application of the total scattering technique at low temperatures. Thus, the cryostat is equipped with windows that allow access to large momentum transfers (±30° in angle) and weakly scattering window material for utilization in combination with large area detectors. A good thermal connection is achieved by keeping the sample in helium gas.

Several modes of operation are provided. Powder samples can be measured by placing them in a multi-capillary holder for 12 samples and moved in the beam via a vertical translation of the sample stick. Individual reflections of single-crystal samples can be investigated with high-angle resolution by rotation of the whole cryostat using the goniometer of the heavy-load sample stage. The reflections are aligned by utilizing a motorized rotation of the sample stick. The ±10° opening angle of the entrance window gives sufficient range for scanning single reflections. This mode is well suited to detect peak splittings or changes in reflection line shape. For the measurement of the total scattering pattern of single crystals a piezo-driven rotation stage mounted on the end of the sample stick is used for sample rotation. It includes a motorized *xy*-translation for moving the sample into the center of rotation. The rotation range around the vertical axis is 200°, sufficient for data sets for 3d-ΔPDF analysis. The utilization of this rotation is limited to temperatures above 5 K. For sample screening at room temperature, a small rotation stage is mounted next to the cryostat. This is well suited for surveying sample quality at room temperature.

#### 2.4.5. Cryostreamer

A cryostreamer for helium and nitrogen is currently in the commissioning phase and specified to reach temperatures down to 28 K with helium at moderate gas consumption of 7.5 l min^−1^. Its main application will be crystallography of small samples. A particular benefit of the cryostreamer versus the cryostats mentioned above is the possibility of container-less measurements, *i.e.* the background signal from windows is avoided and thus does not superimpose and potentially devour very weak signals such as diffuse scattering. Furthermore, the cooling and heating times during sample change are significantly reduced.

#### 2.4.6. 10 T magnet

A 10 T horizontal field magnet cryostat for the investigation of single-crystalline materials under magnetic field is available at the beamline (see Fig. 6 ▸). The split-pair solenoid gives access to the sample in two horizontal directions, with the field direction either parallel or perpendicular to the beam. Both of these pairs of windows for the photon beam have a conical opening angle of ±10°. The variable-temperature insert has a temperature range of 1.8 K to 300 K. The sample can be aligned by a sample stick rotation such that the scattering vector is either parallel or perpendicular to the field. The windows of both the vacuum shield and the variable-temperature insert are made of 500 µm aluminium. Only for single-crystalline samples is a clear distinction of sample scattering from the window powder pattern possible. This limits the sample type that can be investigated with the magnet to single crystals.

#### 2.4.7. Auxiliary equipment

The large diversity of experiments performed at beamline P21.1 often requires specialized equipment that is either part of the beamline equipment pool or is provided by the individual user groups. Custom heating systems can be connected to the temperature controllers and power supplies integrated into the beamline control system to enable remote-controlled temperature variation and readout. Likewise, electrical power can be supplied to *e.g.* a thermoelectric test module or Peltier-element-based cooling chamber.

Long extension cables for different signal standards laid through the chicane between the experimental and control hutch are available to connect equipment at the experiment to the control computers brought by the users and placed beside the beamline control computers for simultaneous operation. Frequently, analog output signals from the user setup are fed into beamline electronics and written into metadata files for each acquired detector image on the PerkinElmer detector; this facilitates the timewise linking between sample state and data. Sample environments in which the sample position is higher than the vertical distance between the beam and surface of the sample stage (315 mm) are installed on an extra sample tower that allows sample-to-beam distances of up to 500 mm. This extra sample tower comprises horizontal and vertical translations, as well as a rotation around the vertical axis if required. A reactor for chemical vapor synthesis of nanoparticles (Schroer *et al.*, 2022 ▸) and a setup for dynamic mechanical testing are examples of sample environments that are too large for the standard diffractometer and required installation on this additional sample stage.

The low-*Q* region relevant for small-angle scattering is accessible when pushing the detector all the way downstream towards the hutch wall, which requires the implementation of a helium-filled flight tube against air scattering. In this setup, the lower limit for *Q* is given by the size of the beamstop behind the exit window of the flight tube at a distance of *ca* 4.5 m and was determined to approximately *Q*_min_ = 0.02 Å^−1^. Two synchronized Varex XRD4343 CT detectors can be used to collect the total scattering and small-angle scattering patterns in a combined range from about 0.02 to 25 Å^−1^ with partial overlap. Due to a cutout in the flight tube covered with Kapton foil, the scattering signal reaching the total scattering detector undergoes negligible absorption. This combined total scattering and small-angle X-ray scattering (SAXS) setup is particularly useful for the simultaneous investigation of atomic structure and morphology of nanostructured samples, *e.g.* nanoparticles growing from solution or under catalytic operation, and polymers during melting and solidification. It has been used in a similar fashion at beamline P07 to monitor the self-assemby of CuPd from precursors to supercrystals (Derelli *et al.*, 2024 ▸).

The sample environment portfolio of the beamline is complemented by additional sample environments available through the sample environment group at PETRA III. These include different devices, *e.g.* for the application of high and low temperature and stress.

### 2.5. Data acquisition and processing

The basic beamline control is performed by *TANGO*, an open-source communication protocol for devices. Command-line and graphical user interfaces (GUIs) found on *TANGO* are *SPECTRA/ONLINE* as well as *SARDANA/SPOCK*.

These GUIs provide all essential functions for data acquisition like triggering signals for detectors, motor scans of single and combined motors, movement of the diffractometer motors in reciprocal space according to the Busing–Levi convention (Busing & Levy, 1967 ▸) and many more. Beamline-specific data acquisition schemes are coded in pytango as *e.g.* continuous scans, multiple detector readouts, or detector and motor readout triggered via a Raspberry Pi Logic Controller module.

For each beam time data are stored on an ASAP3 mass storage which is connected to a general parallel file system (GPFS) (Strutz *et al.*, 2015 ▸). The data are processed on the Maxwell cluster, which is a high-performance computing platform at DESY for photon science data analysis with CPU and GPU accelerated processors. Alternatively, data can be accessed by the Gamma Portal, a web-based interface for data download.

Standard software packages for online data processing and visualization are available via the Maxwell cluster and on beamline computers, including *e.g.* programs for azimuthal integration and calibration of 2D detector geometry. Single-crystal diffraction data are reconstructed by an in-house-developed software package, which is capable of visualizing images recorded with the Pilatus3 X 100K detector mounted on a two-theta arm to survey small portions of reciprocal space for superlattice reflections. The same package is used to reconstruct single-crystal total scattering data for further processing via the 3d-ΔPDF method. This procedure is described in detail by Koch *et al.* (2021 ▸). The data processing of total scattering data of randomly oriented and amorphous materials is performed via a license of *xPDFsuite* or alternatively by the free *PDFgetX3* package. Customized *Jupyter* notebooks are available for online data visualization of azimuthally integrated data including autocorrelation analysis.

For the experimental determination of the elasticity tensor coefficients from thermal diffuse scattering the software packages *TDS2EL2* and *AB2TDS* are provided (Wehinger *et al.*, 2017 ▸). The technique has been successfully applied to a small single crystal of celestite and thiourea where other techniques like *e.g.* ultrasonic measurements, Brillouin scattering and inelastic X-ray or neutron scattering were unsuitable (Girard *et al.*, 2018 ▸; Büscher *et al.*, 2021 ▸).

## 3. Science at P21.1

This section gives an overview of various scientific questions that have successfully been addressed by using the different high-energy X-ray diffraction and scattering techniques available at P21.1. The first two examples are single-crystal studies employing data analysis of diffuse scattering in reciprocal space and in real space via 3d-ΔPDF. They are followed by an investigation on nanoparticles using total scattering and traditional PDF analysis and an X-ray diffraction (XRD) study of bulk alloys during solidification. Finally, data recorded in grazing-incidence total scattering mode are presented. These examples have been chosen to illustrate the wide beamline capabilities and constitute a small selection of science cases from different disciplines and user communities active at P21.1. A complete collection of publications from P21.1 can be found on the beamline’s webpage (https://photon-science.desy.de/facilities/petra_iii/beamlines/p211_high_energy_x_ray_diffraction/index_eng.html).

### 3.1. Local ordering effects in correlated electron materials

Ordering effects on a local scale play a prominent role in correlated electron materials. In a diffraction experiment local order that deviates from the average periodic structure leads to diffuse scattering. Depending on the complexity of the scattering pattern, it can be analyzed either in reciprocal space by modeling a finite-size superlattice or in real space by the PDF method. Here we give examples of experiments where these types of analysis have been applied.

**Figure 7:**
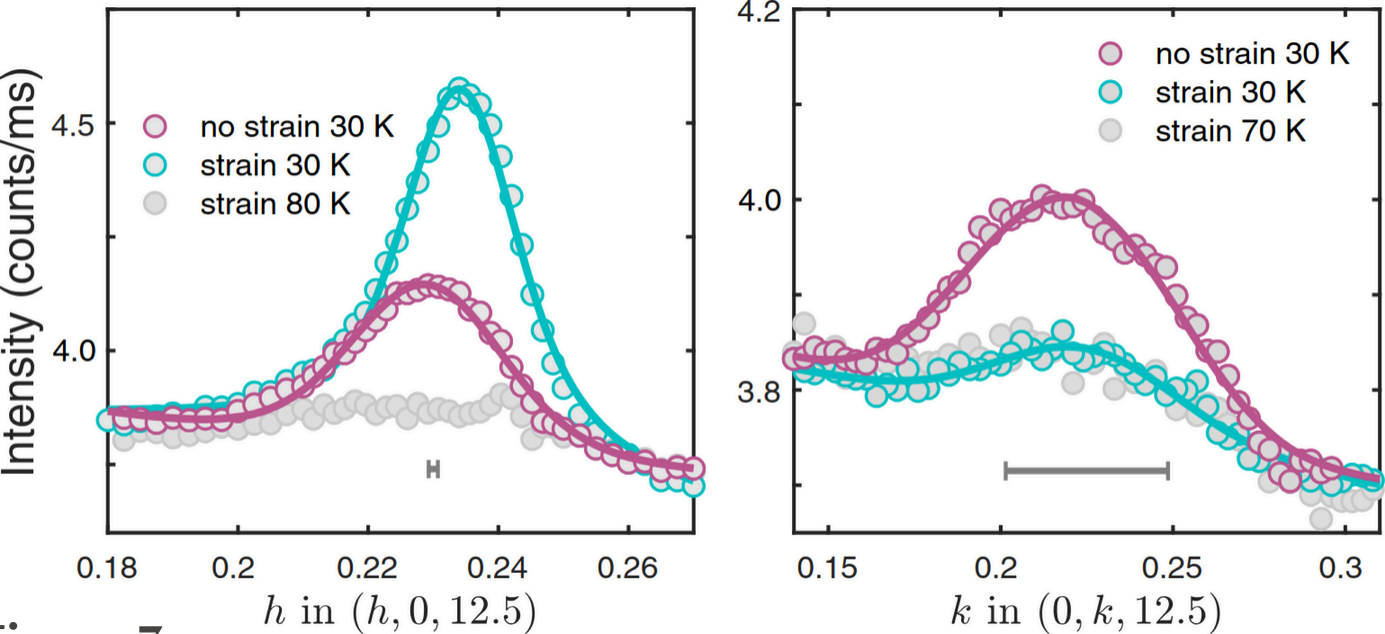
*h* and *k* scan of the charge order reflections at the respective positions *q*_1_ = (0.23, 0, 12.5) (left) and *q*_2_ = (0, 0.23, 12.5) (right) with and without applied strain. The peak under tensile strain at *q*_1_ rises in intensity by a factor of two, the one under compressive strain at *q*_2_ vanishes, indicative of a uniaxial stripe pattern under strain.

It is well known that all cuprate high-*T*_c_ superconductors exhibit charge density wave (CDW) behavior within the pseudogap phase near 1/8 hole doping. A CDW gives rise to superlattice reflections that are attributed to localization of holes in the CuO_2_ planes with concomitant lattice distortions. Whether holes order in a stripe fashion or in checkerboard fashion is an open question. With a high-energy diffraction experiment under uniaxial pressure at beamline P21.1 these two models could be distinguished on a sample of the single-layer cuprate La_1.88_Sr_0.12_CuO_4_ (Choi *et al.*, 2022 ▸). Pressure not only detwins the crystal below the tetragonal–orthorhombic transition but also leads to an increase in the intensity of one of the two equivalent charge order reflections and the dis­appearance of the other, a behavior incompatible with checkerboard order and showing the uniaxial nature of charge order, as shown in Fig. 7 ▸.

**Figure 8:**
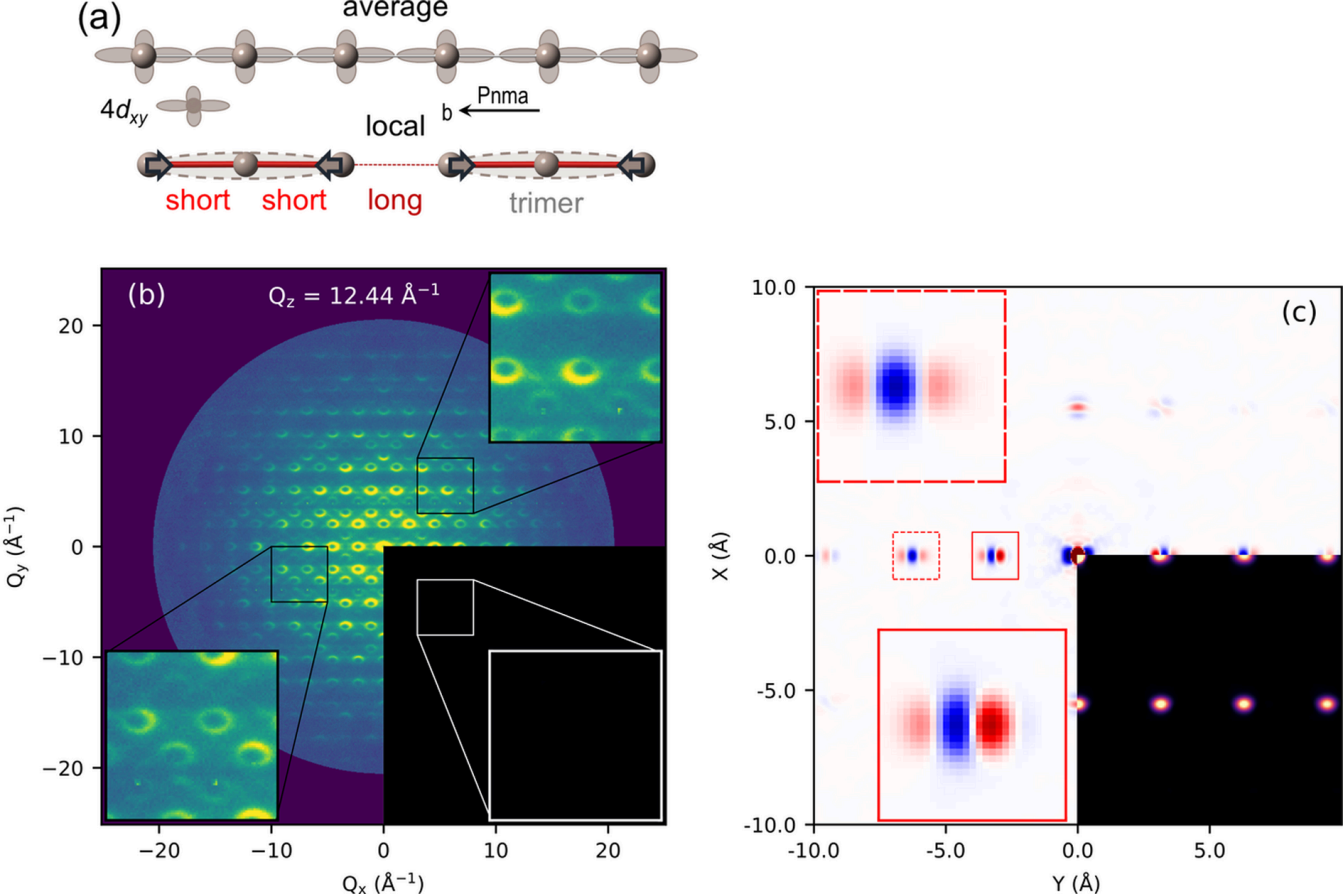
(*a*) Model of the average and local arrangement of Ru atoms in RuP. (*b*) Slice of the diffuse intensity distribution measured from a RuP single crystal in a section between Bragg peaks. (*c*) A cut through the 3d-ΔPDF that shows the most significant features. Ru–Ru nearest-neighbor and next-nearest-neighbor features shown in the enlarged insets are consistent with the presence of trimers along this direction, as depicted in (*a*).

A real-space analysis of local correlations in a RuP single crystal has been determined by employing the 3d-ΔPDF technique combined with data from polycrystalline samples (Koch *et al.*, 2022 ▸). In 3d-ΔPDF the diffuse scattering of a single-crystal sample, caused by local correlations, is separated from the Bragg scattering stemming from the average structure [see Fig. 8 ▸(*b*)]. The Fourier transformation of the diffuse scattering displays the autocorrelation of the deviation of atom positions from their average position [see Fig. 8 ▸(*c*)]. The RuP sample exhibits a pseudogap phase associated with a Fermi surface instability and superconductivity upon doping with *e.g.* Rh. The superconducting phase is linked to the suppression of a nonmagnetic ground state, and its critical temperature, *T*_c_, is maximized around the pseudogap quantum critical point. It is suspected that antiferromagnetic fluctuations mediate superconducting pairs. Formation of local trimers and hexamers might also contribute to the coupling. The powder PDFs derived from data taken at the 28-ID-1 beamline at the National Synchrotron Light Source II (NSLS II) are well described by a *Pnma* model for interatomic distances larger than *r* > 3.9 Å. The model fails at smaller distances and exhibits significant misfits at *r* = 3.1 Å, indicating the presence of a short-range structure distortion, incompatible with the average symmetry. Here a model with monoclinic *P*21/*c* constraints nicely reproduces the local distortion at small radii. The 3d-ΔPDF from the single-crystal total scattering experiment at beamline P21.1 allows a model-independent view of the local distortion. It reveals a complete picture of inter- and intra-rail correlations of Ru–Ru nearest-neighbor and Ru–Ru next-nearest-neighbor pairs. The latter are formed via Ru trimerization, as shown in Fig. 8 ▸(*a*). Interestingly, the two-to-one intensity ratio of the Ru–Ru correlation shown in the lower inset of Fig. 8 ▸(*c*) nicely agrees with the two-to-one ratio of long and short Ru bonds, as illustrated in Fig. 8 ▸(*a*). A high consistency between powder and single-crystal measurements is found regarding distortions and correlation length. These orbital-charge fluctuations in the pseudogap phase might be an important ingredient of the complex phase diagram of binary RuP and their role in superconducting pairing and enhancement of *T*_c_ could be further established by mapping doped compounds.

### 3.2. Growth mechanism and phase formation of nanoparticles during chemical synthesis

**Figure 9:**
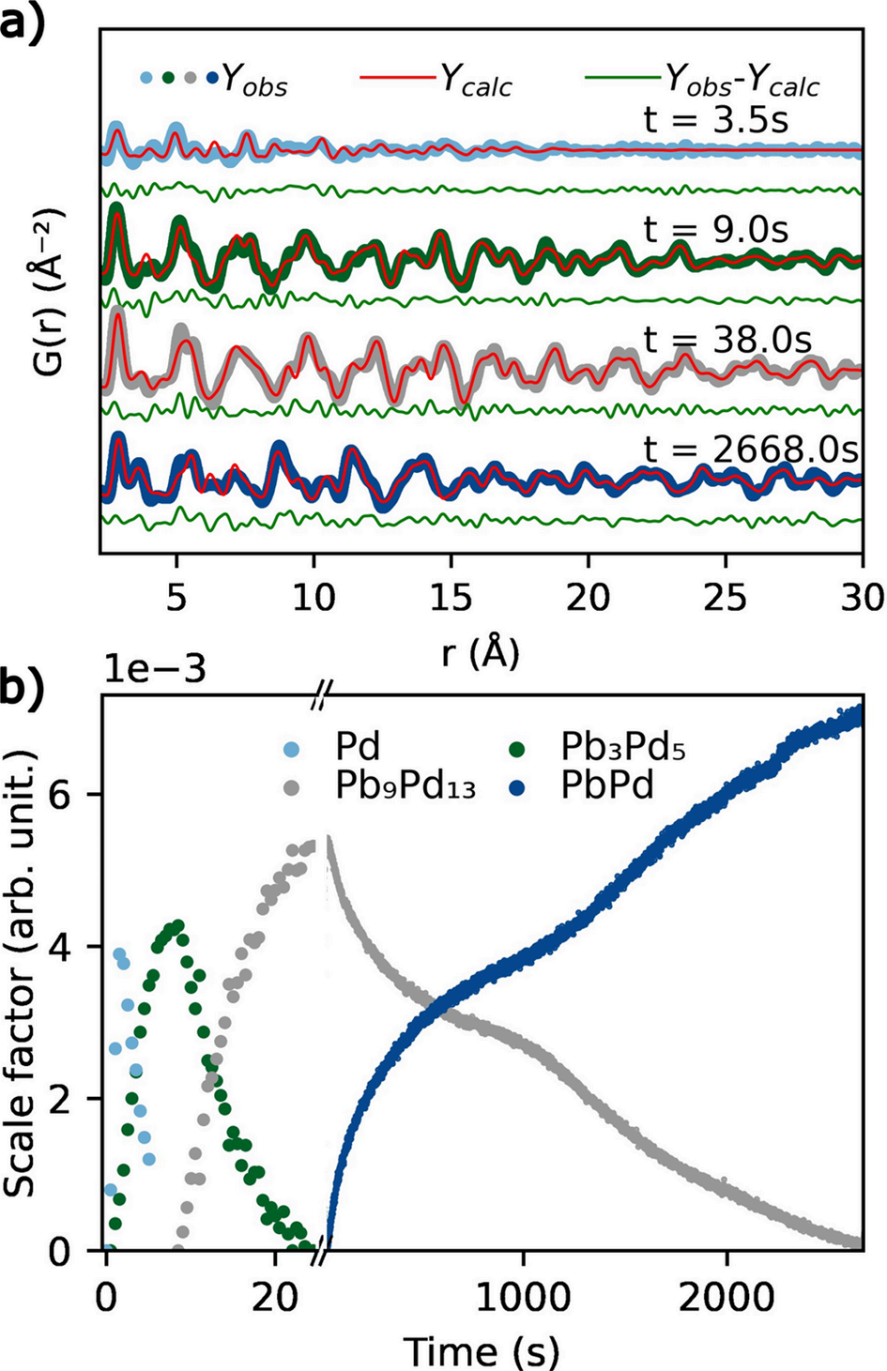
(*a*) PDF patterns of the Pb_*x*_Pd_*y*_ intermetallic reaction mixture at different reaction times at 250°C and 250 bar. (*b*) Refined scale factor for the different phases as a function of time in the same conditions as (*a*).

X-ray total scattering on powders and other bulk-type samples is a versatile technique to monitor chemical processes *in situ*. In the total scattering approach data are collected up to high *Q* of about 20 Å^−1^ or above and Fourier transformed into real space in order to obtain the PDF. *In situ* total scattering studies have proven to give new insights into structural formation processes such as nanoparticle formation by solvothermal synthesis as described by Roelsgaard *et al.* (2023 ▸) and the referenced work therein.This technique was recently employed at P21.1 to follow the incorporation of Pb into Pd nanoparticle intermetallics (Borup *et al.*, 2023 ▸), which paved the way to isolate the phases of different specific intermetallic stoichiometries (Lu *et al.*, 2019 ▸). Pb alloying into Pt has long been observed to enhance its catalytic activity in various reactions. However, the presence of multiple stoichiometric phases and interdependency between substitution and size effect had made it difficult to optimize the synergy between Pb and Pt. The reaction between the two acetylacetonates Pd(acac)_2_, Pt(acac)_2_ and ethylene glycol under solvothermal conditions was followed as a function of time and temperature, inside a pressurized cell at about 250 bar. By measuring total scattering patterns with a time resolution of 0.5 s and a *Q*_max_ of 33 Å^−1^, the nucleation and growth mechanism could be retrieved through PDF analysis. Pure Pd nanoparticles nucleate and grow at the early stages of the reaction, up to 3.5 s after reaching 250°C, as observed from the PDF [Fig. 9 ▸(*a*)]. Pb is then absorbed and diffuses into the Pt particles until it forms stoichiometric phases in the sequence Pb_3_Pd_5_ → Pb_9_Pd_13_ → PbPd, as shown by the sequentially refined scale factors of the multiple phases [Fig. 9 ▸(*b*)]. For this analysis, the representation of the crystallite structure in the PDF is superior to the scattering patterns. The latter contain only few Bragg reflections that are very broad in the early stages of nucleation and growth, while the PDF reveals the atomic structure of the crystalline domains independent of their size. In *ex situ* syntheses in the laboratory, the Pb_3_Pd_5_ and Pb_9_Pd_13_ phases could be isolated by limiting the diffusion rate of Pb into Pd at lower temperatures, *i.e.* 150 and 200°C, respectively. For nanoparticles of these stoichiometries, the study of their electrocatalytic properties towards hydrogen evolution resulted in comparable stability and overpotential characteristics as in pure Pd.

### 3.3. Nucleation and phase propagation during solidification of alloys

**Figure 10:**
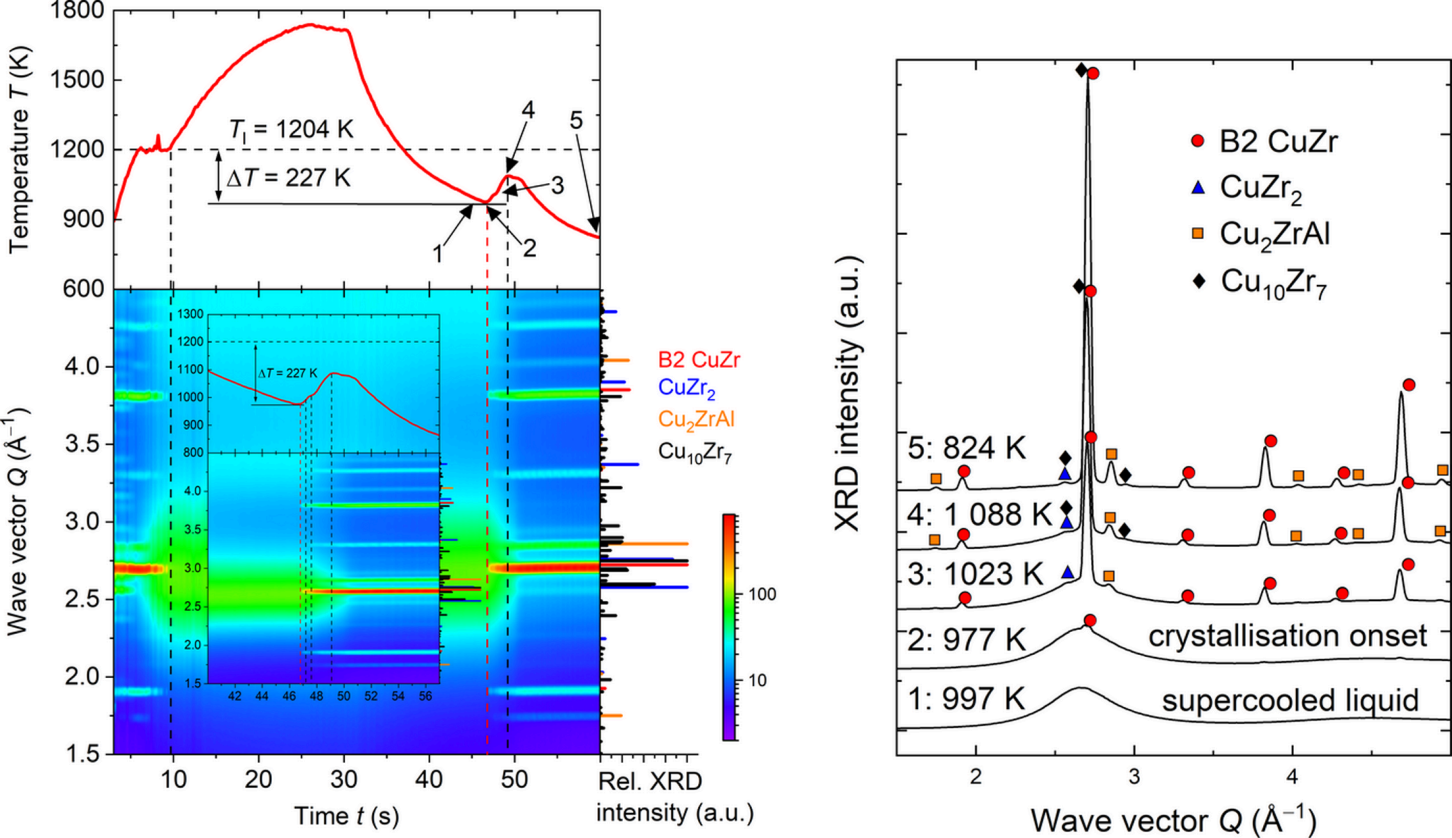
Left: time–temperature curve *T*(*t*) and 2D contour plot of the XRD intensities measured from a Cu_47.5_Zr_47.5_Al_5_ alloy during heating–cooling processing in the IFW Dresden EML facility at P21.1. *T*_l_ is the liquidus temperature; Δ*T* = 232 K is the melt undercooling right before crystallization onset. Right: selected XRD patterns elucidating the phase formation sequence during crystallization of the undercooled liquid. The numbers correspond to those marked on the *T*(*t*) curve. The symbols denote the position of the characteristic peaks of the identified phases.

Non-equilibrium solidification of undercooled liquid metals and high-temperature solid-state phase transformations in metallic alloys have been studied by *in situ* diffraction by the group of Ivan Kaban from Leibniz-IFW Dresden. For these experiments, a mobile electromagnetic levitation (EML) facility has been custom-built for measurements at high-energy X-ray diffraction beamlines at PETRA III. Spheroid-like metallic samples of 6–7 mm diameter are levitated in an induction coil situated inside the experimental chamber under inert gas atmosphere. During heating and/or cooling of the sample, depending on applied electric current and cooling gas jet, the induced changes are recorded using a high-speed video camera, optical pyrometer and fast 2D X-ray detector. The pyrometer and detector signals are synchronized, enabling mapping of the time–temperature–structure dependencies, as demonstrated for the Cu_47.5_Zr_47.5_Al_5_ metallic glass-forming alloy (Orava *et al.*, 2021 ▸) in Fig. 10 ▸. Electromagnetic levitation in a high-pure inert gas atmosphere allows sample contamination to be avoided, which usually occurs in crucible-based high-temperature experiments. Furthermore, it essentially reduces the possibility of heterogeneous nucleation in the melt during cooling. Therefore, a large undercooling Δ*T* of the melt below the equilibrium liquidus temperature *T*_l_ can be achieved. Due to the use of the fast 2D X-ray detector, operating at the frequency of ≤250 Hz (Table 3 ▸), and excellent signal-to-noise ratio, high-quality XRD patterns revealing the onset of crystallization and phase formation or transformation sequences can be reliably determined. Recently, this experimental setup has been successfully used at P21.1 for studies of solidification and phase transitions in medium- and high-entropy alloys such as CrFeNi, CoCrNi, CoCrFeNi (Andreoli *et al.*, 2021*b* ▸), Al_*x*_CoCrFeNi (Andreoli *et al.*, 2022 ▸), NbTiVZr (Andreoli *et al.*, 2021*a* ▸) and the compositionally complex alloy Al_20_Cr_20_Fe_35_Ni_20_Ti_5_ (Wolff-Goodrich *et al.*, 2022 ▸).

### 3.4. Local atomic structure of amorphous and nanocrystalline thin films

**Figure 11:**
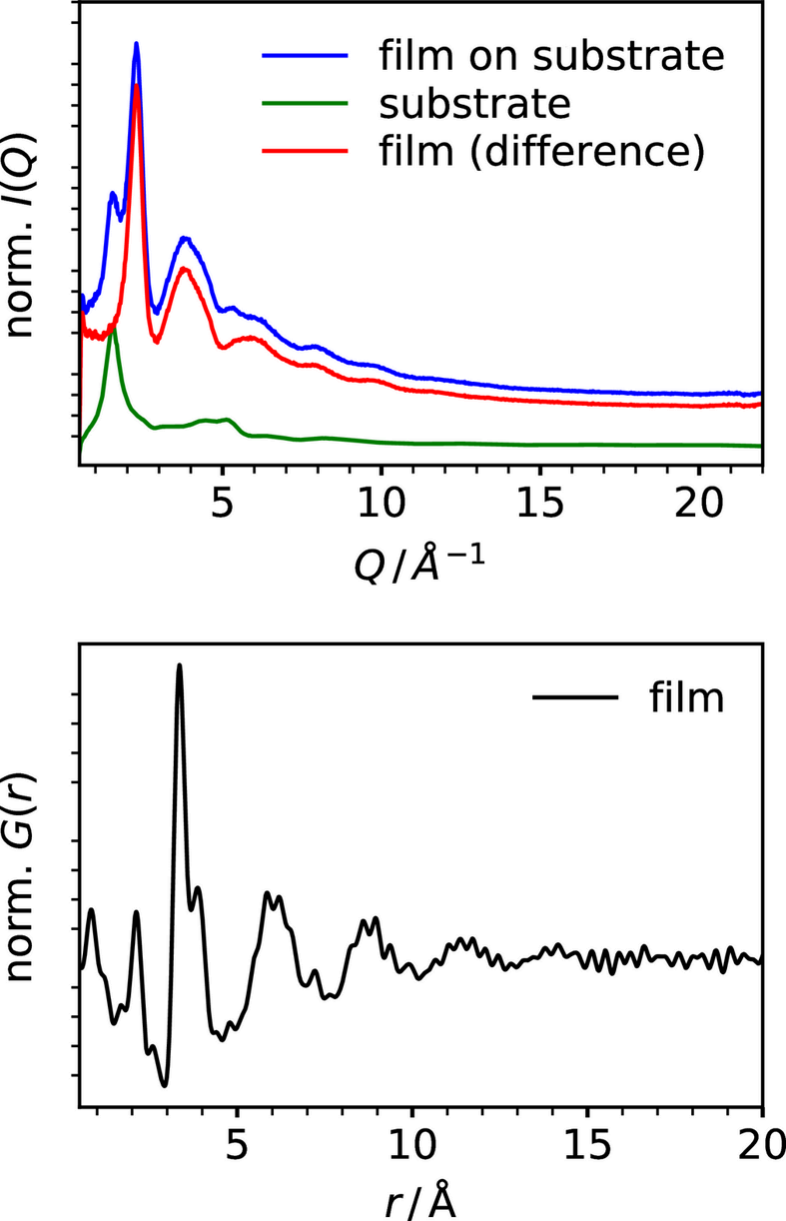
(Top) Grazing-incidence total scattering patterns of a sample film on a substrate, the blank substrate and the difference from subtracting the scaled background from the sample data. The sample is a 30 nm HfO_2_ thin film deposited by sputtering at room temperature onto fused silica; the substrate is a blank piece of the same fused silica. The data were collected at P21.1 with a focused beam at an energy of 101 keV on the Pilatus3 X CdTe 2M detector positioned at about 300 mm downstream of the sample. (Bottom) PDF of the HfO_2_ thin film obtained by Fourier transformation of the film signal as the difference between the sample and the substrate from the top panel.

Beyond the traditional application of total scattering and PDF analysis to bulk-type systems measured by powder diffraction methods, the technique has more recently been advanced to investigate the local structure of thin films (Jensen *et al.*, 2015 ▸; Stone *et al.*, 2016 ▸; Dippel *et al.*, 2019 ▸). The particular difficulty of obtaining high-quality total scattering data from thin films lies in the small amount of sample being spread out in 2D but confined to typically below 1 µm in the third dimension. In addition, a thin film is usually deposited on a substrate that is thicker by a factor of at least 1000 (nanometre film thickness versus micro- to millimetre thickness of the support). Though transmission measurements with the incidence beam perpendicular to the film surface are experimentally straightforward, they provide an unfavorable signal-to-background ratio due to the substrate contribution and thus effectively restrict the detection limit to thicker films, typically at least several tens to hundreds of nanometres. By contrast, surface diffraction type measurements in grazing incidence (GI) yield enhanced surface sensitivity of the film regardless of the thickness of the substrate. In our recent work performed at beamline P07, we demonstrate the benefits of GI total scattering to determine the short-range order of diverse thin film systems with thicknesses down to a few nanometres (Dippel *et al.*, 2019 ▸; Roelsgaard *et al.*, 2019 ▸). With its newly available focusing option, P21.1 now offers the added capability for total scattering at GI, *i.e.* micro-focused high-energy X-rays in combination with a large, fast area detector. Fig. 11 ▸ shows the PDF extracted from a 30 nm film of HfO_2_ sputter-deposited on a fused silica substrate to exemplify P21.1’s GI total scattering performance. The first two sharp PDF peaks at 2.1 Å and 3.4 Å represent the nearest-neighbor Hf–O distance and the next-nearest-neighbor Hf–Hf distance in the structure. Above 5 Å, the broad maxima indicate a large degree of disorder until the correlations die out into noise at approximately 13 Å. This lack of long-range ordering is common for many oxide layers sputtered at room temperature. When annealing the as-deposited HfO_2_ films at elevated temperatures, they crystallize into one or a mixture of polymorphs, mostly the monoclinic and/or tetragonal phases. Under certain conditions, however, the non-centrosymmetric orthorhombic phase of space group *Pca*2 also forms which gives rise to ferroelectricity in HfO_2_-based films and devices (Böscke *et al.*, 2011 ▸).

Various parameters affect the ferroelectric performance such as the stoichiometry, *i.e.* oxygen vacancies and dopants, as well as during deposition and post-deposition treatment. Berg *et al.* described the complex interdependence of crystallographic phase and electrical properties in their sputter-deposited HfO_2_ films (Berg *et al.*, 2023*a* ▸; Berg *et al.*, 2023*b* ▸), similar to the one for which we are presenting data here, emphasizing the importance of understanding the structure–function relationship to tune ferroelectricity. Characterizing the crystalline state of thin films is feasible using suitable laboratory-source X-ray diffraction instruments, but it fails when a high-*Z* material is deposited on top of the ferroelectric layer and absorbs the incoming X-rays, for instance a platinum electrode that supposedly catalyzes the evolution of the ferroelectric phase, as described by Berg *et al.* (2023*b* ▸). The penetration power of high-energy X-rays overcomes these limitations as it enables one to probe a stack of layers, as described *e.g.* by Dippel *et al.* (2020 ▸). In addition, GI total scattering is an indispensable tool to obtain information on the short-range atomic ordering. Here, the similarity of the local chemical environment to one of the polymorphs like the monoclinic phase, as in the example shown in Fig. 11 ▸, possibly favors the formation of one or the other polymorph during different thermal treatments varying in atmosphere, heating rate *etc*. The investigation of such dependencies is now possible at beamline P21.1, addressing thin film communities that rely on chemical and physical fabrication techniques, such as atomic layer deposition (ALD), sputtering, and spin-coating or dip-coating. A recent study on amorphous doped indium tin oxide layers shows that PDF analysis is viable for film thicknesses <10 nm, as typically targeted *e.g.* in ALD (Büschges *et al.*, 2024 ▸). Furthermore, the study of aluminium oxide formation during the anodization of aluminium in an electrochemical cell reported by Magnard *et al.* (2025 ▸) demonstrates the beamline capabilities for time-resolved GI total scattering and, to our knowledge, constitutes the first *in situ* total scattering experiment on amorphous thin films directly growing on top of a single-crystalline substrate.

## 4. Conclusions and perspectives

In conclusion, P21.1 is a beamline for the determination of structural properties of samples in the field of physics and chemistry. Samples in the form of powders, single crystals and thin films can be investigated under various *in situ* conditions, ranging from temperature and magnetic field to chemical environments.

The capabilities of the beamline regarding optics, sample environments, detectors and data processing and analysis tools are constantly upgraded, in order to make more efficient use of the source and to expand the user community. For instance, the use of multilayers for harmonic suppression will increase the flux on the sample at 53 and 86 keV by more than a factor of five and make these energies much more attractive for total scattering studies on *e.g.* weakly scattering systems such as those composed of light elements. The constant oversubscription of the Pilatus 2M detector calls for expansion of the pool of large area hybrid-pixel detectors that allow measurements requiring high dynamic range and low noise. Efforts have been made to expand the beamline’s capabilities for grazing-incidence measurements on thin films towards non-randomly oriented samples and towards low-temperature applications. Furthermore, sample environments are being expanded regarding temperature range and sample throughput as much as possible, responding to user demand. Online data visualization and processing is subject to ongoing development.

The upgrade of the storage ring to PETRA IV, a lattice with six bent achromats, will dramatically reduce the horizontal emittance and consequently make beam focusing much more efficient. At the same time, beamlines will be upgraded or rebuilt. The new lattice means that beamline P21 stays in the same place and thus will be able to operate from day one after the shutdown.

## Figures and Tables

**Table 1:** List of beamline front-end and U29 undulator parameters; *K* is the dimensionless undulator strength parameter

Source size (FWHM) (V × H)	11.5 µm × 332.5 µm
Divergence (FWHM) (V × H)	4.9 µrad × 16.7 µrad
Undulator length	2 m
Period length	29 mm
No. of periods	67
Maximum *K*	2.2

**Table 2:** List of beamline photon-beam parameters with the single-bounce monochromator

Material	Si
Reflections	111, 220 or 311
Scattering plane	Horizontal
Crystal thickness	2.5 mm
Bending radius	10 m to ∞ to −10 m
Cooling medium	In–Ga/water
Energy	53 keV, 86 keV or 101 keV
Band pass Δ*E*/*E*	1 × 10^−4^ to 5 × 10^−3^
Maximum flux density	5 × 10^11^ photons s^−1^ mm^−2^ at 101 keV
	1.1 × 10^12^ photons s^−1^ mm^−2^ at 53 keV†
Beam size (FWHM V × H)	2 µm × 80 µm to 1 mm × 1 mm

† Including harmonic suppression by the secondary monochromator.

**Table 3:** Detectors available for experiments at beamline P21.1 and their properties

Detector	Area (mm)	Pixel size (µm)	Max. frame or count rate (s^−1^)
Flat-panel detectors			
PerkinElmer XRD1621†	409.6 × 409.6	200 × 200	15
Varex XRD 4343CT†	432 × 432	150 × 150	15‡

Hybrid-pixel detectors			
Pilatus3 X CdTe 2M‡	253.7 × 288.8	172 × 172	250§
Pilatus3 CdTe 100K	83.8 × 33.5	172 × 172	500
Eiger2 X CdTe 4M‡	155.1 × 162.2	75 × 75	560§

Point detectors			
Amptek XR-100-CdTe	5 × 5	N/A	10^5^
Amptek XR-100-Cr Si-PIN	5 × 5	N/A	10^5^
FMB NaI / LaBr3	Ø30	N/A	10^6^

† Composed with CdI:Tl columnar scintillator.

‡ Pool device.

§ Higher frame rates are achievable by pixel binning or with reduced dynamic range.

**Table 4:** Sample environments for heating and cooling available for experiments at beamline P21.1 RT = room temperature.

Sample environment	Sample type	Temperature range
Resistive heating plate (standalone)	Powder, liquid, thin film	RT to ≤1000°C
Resistive heating plate (in scattering chamber)	Powder, liquid, thin film	RT to ≤1000°C
Hot air blower in splash box	Powder, liquid	RT to ≤800°C
Displex cryostat in Eulerian cradle	Single crystal	10 to 320 K
Displex cryostat in cryochamber	Powder, single crystal	30 to 320 K
Helium bath cryostat	Powder, single crystal	1.8 to 300 K
Magnet cryostat (*H* ≤ 10 T)	Single crystal	1.8 to 300 K
Cryostreamer	Single crystal	28 to 320 K
